# Quantitative Evaluation of a Telerobotic System for Vascular Ultrasound Measurement on a Short Arm Human Centrifuge

**DOI:** 10.1007/s12217-020-09850-8

**Published:** 2021-01-23

**Authors:** Timo Frett, Guido Petrat, Michael Arz, Carole Leguy

**Affiliations:** 1 German Aerospace Center (DLR), Institute of Aerospace Medicine, 51147 Cologne, Germany; 2 Institute of Measuring and Sensor Technology, Ruhr West University of Applied Science, Mülheim an der Ruhr, Germany

**Keywords:** Telerobotic, Artificial gravity, Ultrasound imaging, Vascular response, Space physiology

## Abstract

Artificial Gravity generated by Short Arm Human Centrifuges is a promising multi-system countermeasure for physiological deconditioning during long duration space flights. To allow a continuous assessment of cardiovascular hemodynamics during centrifugation, a telerobotic robotic system holding an ultrasound probe has been installed on a Short Arm Human Centrifuge. A feasibility study was conducted to define the use capabilities and limitations of such a novel method. The objective of this study is to estimate the reproducibility and precision of remotely controlled vascular ultrasound assessment under centrifugation by assessing peripheral vascular diameter and wall distension. Four repeated centrifugation runs of 5 min, with 2.4 g at feet level, were performed including a 15 min rest between each run for a group of eight healthy male volunteers. Vascular diameter and distention were assessed for the common carotid artery (CCA) and the femoral artery (FA) by ultrasound imaging using a 10 MHz linear array probe (Mylab1, Esaote). Ultrasound measurements were consecutively performed: a) by an expert user in hand-held mode in standing as well as supine position, b) using the telerobotic arm without centrifugation as baseline and c) using the telerobotic arm during centrifugation. Vascular responses were compared between baseline and under centrifugation. Inter-, intra-registration and group variability have been assessed for hand-held and remotely controlled examination. The results show that intra-registration variability, *σ*_h_ , was always smaller than inter-registration variability, *σ*_m_, that is in turned smaller than the inter-subject variability *σ*_g_ (*σ*_h_ < *σ*_m_ < *σ*_g_). Centrifugation caused no significant changes in CCA diameter but a lower carotid distension compared to manual and robotic ultrasound in supine position (*p* < 0.05). Femoral diameter was significantly decreased in hypergravity compared to robotic sonography without centrifugation. A good reproducibility and precision of the remotely controlled vascular ultrasound assessment under centrifugation could be demonstrated. In conclusion, arterial wall dynamics can be precisely assessed for the CCA and femoral artery during centrifugation using a telerobotic ultrasound measurement system. Potential improvements to further enhance reproducibility and safety of the system are discussed.

## Introduction

In preparation for the next phase of manned long term spaceflight exploration a more profound understanding of cardiovascular responses to gravitational load is crucial. While standing upright on Earth, a head-to-foot gravitational force results in regular fluid shifts to and from the head. In microgravity this process alters and elicits a decrease in cardiac output that can induce post-spaceflight orthostatic intolerance which remains a major issue for astronauts (Lee et al. 2015; Gunga et al. 2016).

Artificial Gravity generated by short arm centrifugation is considered to counteract space flight-induced cardiovascular deconditioning as it induces a hydrostatic pressure column along the body long axis to simulate Earth’s gravity (Stenger et al. 2012; Clément and Pavy-Le Traon 2004; Iwase et al. 2007; Linnarsson et al. 2015). Recent design models recommend intermittent short-radius centrifugation (radius < 10 m) for exercise activities during long term missions that will result in a significant gravity gradient along the body longitudinal axis (Clement 2017; Simón et al. n.d.). A better knowledge on cardiovascular responses to a steep gravity gradient is essential to develop future countermeasures based on acritical gravity.

Using functional ultrasound imaging, it is possible to assess non-invasively and in real time, arterial diameter and wall distension at peripheral arteries as the Common Carotid Artery (CCA) or the femoral artery (FA). These methods, based on radio-frequency signal techniques, are recognized as the gold standard in arterial wall dynamics measurement. The automatic detection of the artery wall directly from radio-frequency signals has the advantage of being more reproducible and user-independent than manual detection by the end user (Leguy et al. 2009; Meinders and Hoeks 2004; Brands et al. 1999). An accurate and stable probe positioning is crucial for such measurements and must be continuously adjusted by the sonographer. During centrifugation, the sonographer must remain remote from the subject in the control room. Therefore, only a telerobotic system can allow such measurements. While telerobotic systems have been developed for various clinical applications (Dogangil et al. 2010; Marescaux and Rubino 2003; Evans et al. 2018) their employment on a human centrifuge requires specific safety measures. For this purpose, a telerobotic ultrasound system for vascular sonography has been built on the DLR Short Arm Human Centrifuge (Frett et al. 2014a) that can be used in hypergravity conditions up to +6 g. The objective of the study is to estimate the reproducibility and precision of a remotely controlled real-time assessment of arterial diameter and wall distension during centrifugation using functional telerobotic ultrasound imaging. To our knowledge, haemodynamic measurements using ultrasound functional imaging during centrifugation were never been performed on humans. We compared measurements of carotid and femoral diameter during centrifugation using the telerobotic ultrasound system with hand-held and telerobotic measurements before centrifugation. Results were analyzed for inter-subject in terms of inter-registration variability as well as intra-measurement variability.

## Methods

### Study Subjects

In accordance with the ethical committee North Rhine, Germany (Communications Reference Number 2014258) 9 healthy and non-smoking male subjects were recruited. Their average age was 30 ± 5 years, their average weight 80 ± 9.2 kg, and their average height 182 ± 4 cm. All subjects passed standard medical screening for the centrifuge experiments and having provided written informed consent.

### Telerobotic Ultrasound

Ultrasound measurements were recorded while subjects lying supine on DLR’s Short Arm Human Centrifuge (SAHC) (Frett et al. 2016; Frett et al. 2014a; Frett et al. 2014b). A point-of-care ultrasound system (ESAOTE MyLabOne, Italy) equipped with a 10 Mhz vascular probe was customized by the manufacturer to be remotely controllable from the centrifuge control room and fixed safely on the centrifuge.

**Fig. 1:**
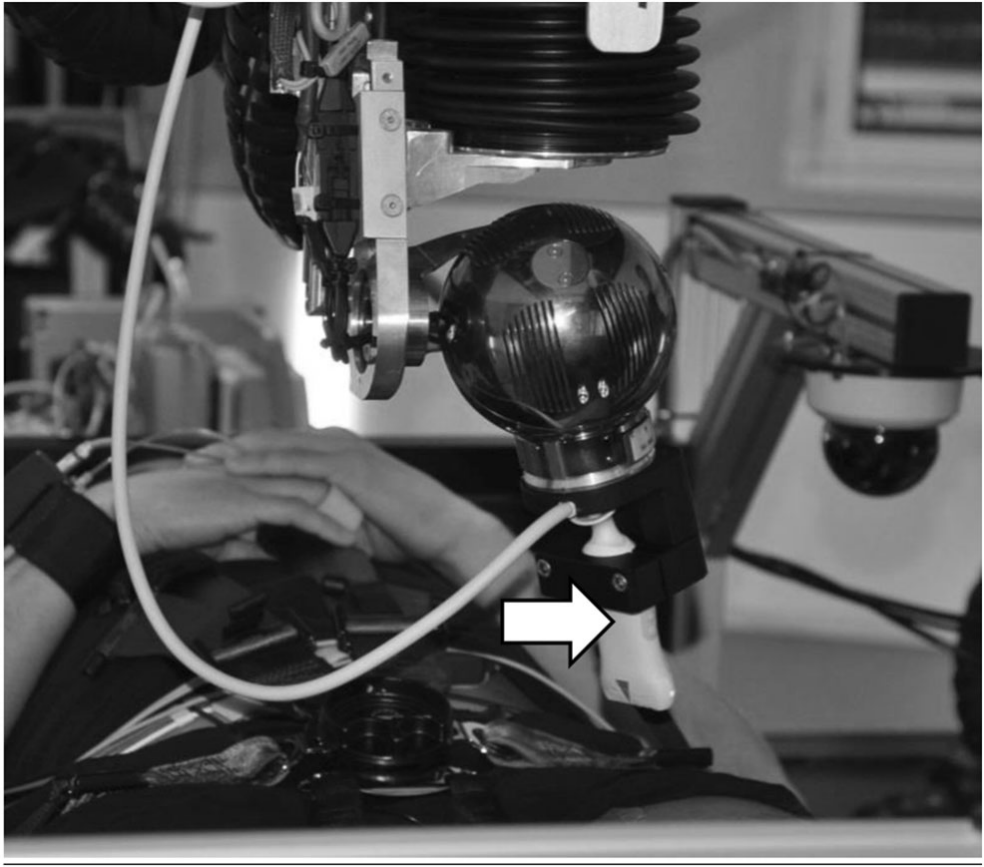
Remotely controlled robotic system with vascular probe mounted at the tip (arrow)

**Fig. 2:**
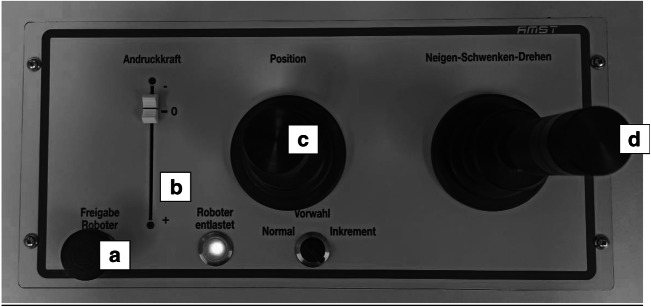
Remote control panel for the robotic ultrasound system. **a** Safety button **b** Slide control to adjust probe pressure on subject’s skin. Maximal applicable pressure with the slide control can be limited in the software between 1 and 100 N) **c** 2-dimensional linear axes joystick **d** 3-dimensional rotatory control

The ultrasound scanner settings are remotely controllable from the sonographer and a 1:1 reproduction of the normal scanner display was carried to a monitor in the control room. The ultrasound probe is mounted on the tip of the telerobotic arm that was first positioned above the subject (see Figs. 1 and 2). By using a control panel in the centrifuge control room, the sonographer can adjust the probe position in all 6 degrees of freedom within safety limits also while the centrifuge is spinning. The robotic arm has a position accuracy of ±1 mm. Pressure on the subject’s skin is controlled by a pressure system that limit the contact pressure from 1 to a maximum of 100 N. This pressure was carefully adjusted depending on the probe position and overall limited to 20 N (Frett et al. 2014a). During centrifugation, the current probe position was monitored by two video cameras on different angles.

On the centrifuge, subjects were secured wearing a 6-point safety belt (Schroth, Germany) and non- invasively monitored by an attending physician (electrocardiogram, manual blood pressure and oxygen saturation). Subjects were told not to move during the measurement and were blindfolded to reduce the risk of motion sickness symptoms.

### Protocol

All subjects participated in one experiment session on the Short-Arm Human Centrifuge at the DLR Institute of Aerospace Medicine, Cologne (Germany). An anamnesis and familiarization with the centrifuge´ robotic arm were performed prior to the experiment. Baseline measurements included height, body weight, distance head (apex) to CCA (around 2 cm below chin) were assessed.

**Fig. 3:**
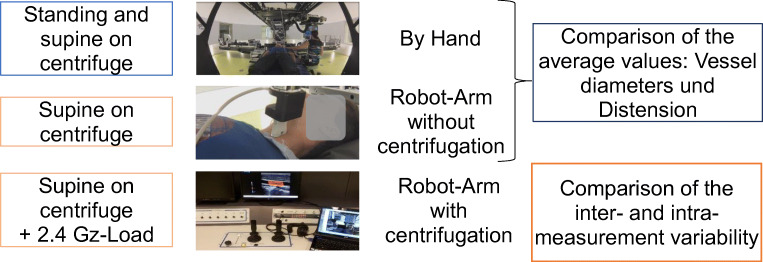
Measurement during all four study conditions: manual sonography standing and in supine position, robotic sonography with and without centrifugation in supine position

Before centrifugation optimal probe position for sonography of CCA and FA were marked on the subject’s skin with medical tape. Baseline measurements of the carotid and femoral mean diameter *D* and vessel wall distension, *∆D* (difference between systolic and diastolic diameter), were recorded in randomized order (Fig. 3):

- by hand in supine position, condition: ‘hand-supine’,
- with the ultrasound probe attached at the robotic arm from supine position without centrifugation, condition: ‘robotic-static’, and,
- with the remotely controlled robotic arm during centrifugation at +2.4Gz at feet: condition: ‘robotic-centrifugation’.

**Fig. 4:**
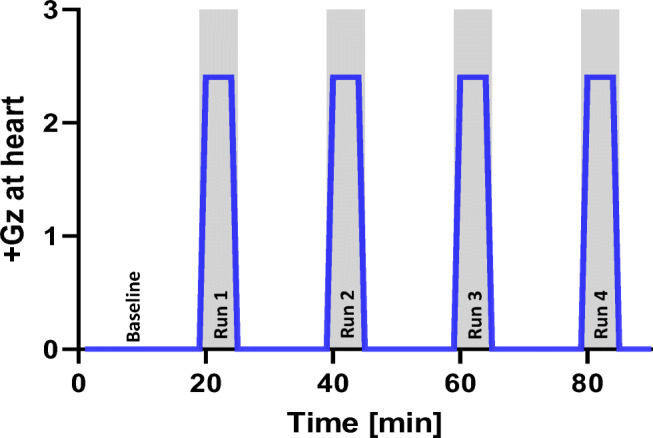
Centrifuge profile with four sessions of artificial gravity at +2.7Gz @ heart level

Centrifuge speed and subject axis position was calculated for a g-load of +2.4 *Gz* at feet level and + 1.0 Gz at the heart level. Resulting *+Gz*-load at heart and carotid level were recorded depending on subject’s body height and measured distances CCA-Apex and CCA-heart. The centrifuge profile consisted of four hyper-G phases with constant individual RPM (rotation per minute). Mean spin rate of the centrifuge was therefore 29 ± 0.4 rpm with +0.9 *Gz* at Carotis, +1.0 *Gz* at heart and + 2.4 *Gz* at feet. To ensure subjects orthostatic stability at the combined high g-load and steep gradient, each centrifuge run was limited to 5 min. Assessment of arterial diameter and wall distention from carotid and femoral artery were randomized between the subjects Fig. 4.

### Statistical Analysis

The variability of the assessed vessel diameter and wall distension was investigated under the different conditions. We differentiated, for each condition, among the

- intra-registration variability, *σ*_h_, between the consecutive heartbeats within single measurements, (Eq. 1),
- inter-registration variability *σ*_m_ between repeated measurements, (Eq. 2), and,
- inter-subject variability *σ*_g_ within the volunteer of the group (Eq. 3).

The intra-registration variability *σ*_h_ is defined as:

1 $$ {\sigma}_h=\sqrt{\frac{\sum_v{\sum}_m{\sum}_b{\left({X}_{v,m,b}-{\overline{X}}_{v,m}\right)}^2}{\sum_v{\sum}_m\left({b}_{v,m}\right)-{n}_m}} $$

with *X*_*v*, *m*, *b*_ a parameter representing either the assessed average diameter (*D*) or the vessel wall distension (*∆D*) for the volunteer *v*, in measurement *m* at heart-beat *b*. The average parameter for the measurement *m* of volunteer *v* is given by${\overline{X}}_{v,m}$. The number of beats for a measurement *m* for the volunteer *v* is given by *b*_*v*, *m*_ and the total number of measurements by *n*_*m*_.

The inter-measurement variability *σ*_*m*_ is defined as:

2 $$ {\sigma}_m=\sqrt{\frac{\sum_v{\sum}_m{\left({X}_{v,m}-{\overline{X}}_v\right)}^2}{\sum_v\left({m}_v\right)-v}} $$

with *X*_*v*, *m*_ the parameter value for the volunteer *v* and measurement *m*, ${\overline{X}}_v$ the average parameter for the volunteer *v*. The number of measurements for a volunteer *v* is given by *m*_*v*_, and, the number of volunteers by *v*.

The group-variability (or inter-subject variability) *σ*_*g*_ is given by:

3 $$ {\sigma}_g=\sqrt{\frac{\sum_v{\left({X}_v-\overline{X}\right)}^2}{v-1}} $$

with *X*_*v*_ the parameter average for the volunteer *v* and $\overline{X}$ the group average.

Results for each condition were analyzed using paired t-tests to compare each condition. Mean values were reported with standard deviation. All statistical tests were conducted using IBM SPSS version 21 (IBM Corp., USA) with α < 0.05 indicating significance.

## Results

Two subjects were excluded, one due to pre-syncopal symptoms during centrifugation, and one other because of technical problems. From the remaining seven subjects, complete data-sets (with valid ultrasound hemodynamic measurements during centrifugation) have been obtained from six subjects for the carotid artery with a success rate of 71%, and, four for the femoral artery with a success rate of 57%.

**Fig. 5:**
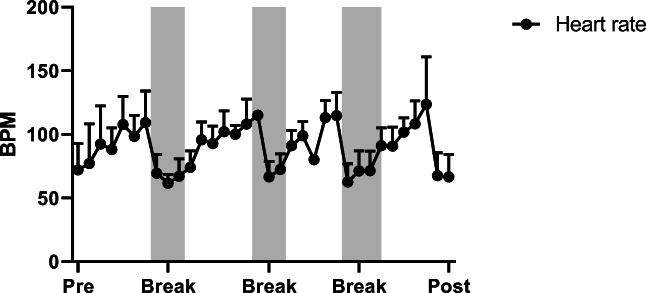
Average values for heart rate, systolic and diastolic blood pressure with standard deviation during centrifugation. Baseline measurements were taken before centrifugation (pre) and during the experiment including breaks between centrifuge runs until end of protocol (post)

**Fig. 6:**
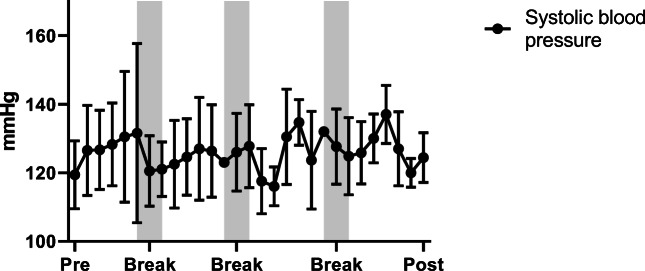
Average values for heart rate, systolic and diastolic blood pressure with standard deviation during centrifugation. Baseline measurements were taken before centrifugation (pre) and during the experiment including breaks between centrifuge runs until end of protocol (post)

**Fig. 7:**
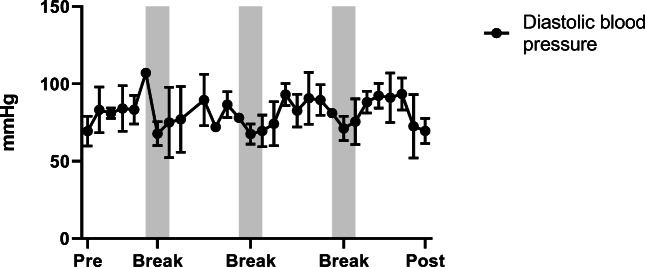
Average values for heart rate, systolic and diastolic blood pressure with standard deviation during centrifugation. Baseline measurements were taken before centrifugation (pre) and during the experiment including breaks between centrifuge runs until end of protocol (post)

Mean values for heart rate, systolic and diastolic blood pressure are 74 ± 20.17 bpm, 119.4 ± 9.27 mmHg and 69.4 ± 9.0 mmHg before centrifugation (rest supine), and, 68 ± 18.0 bpm, 122.6 ± 5.9 mmHg and 69.1 ± 7.5 mmHg after centrifugation (recovery), with no significant differences. During centrifugation, mean values are 95 ± 15 bpm for heart rate, 126.4 ± 5 mmHg for systolic and 83.8 ± 8.6 mmHg for diastolic blood pressure (Figs. 5, 6 and 7) with significant increase of heart rate, systolic and diastolic blood pressure (*p* < 0.05). No significant differences for heart rate and blood pressure are obtained between the four centrifuge runs.

**Fig. 8:**
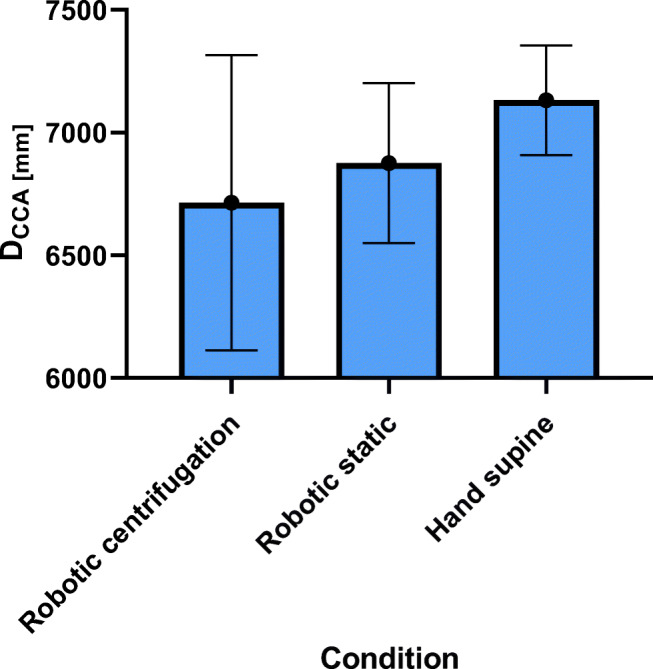
Carotid diameter (*D*_*CCA*_) for all four conditions (hand supine, robotic static, hand vertical, robotic centrifugation)

**Fig. 9:**
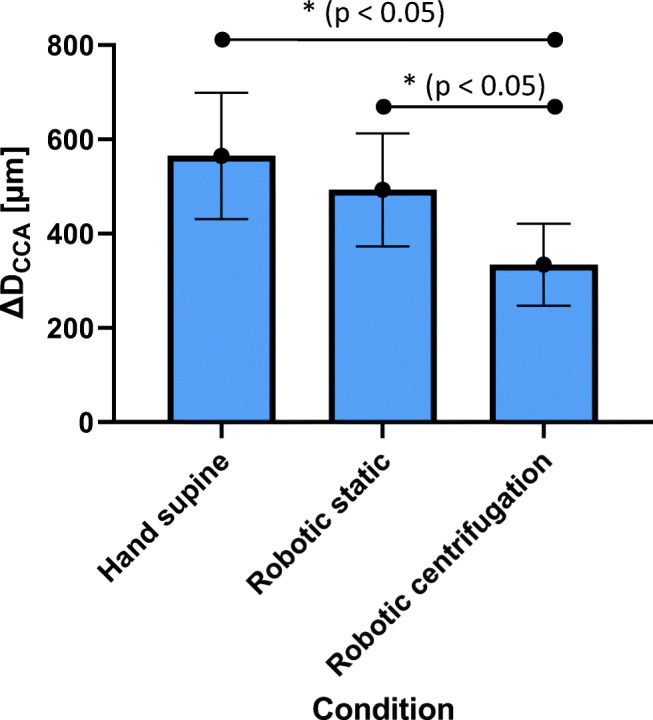
Carotid distention (*∆D*_*CCA*_) for all four conditions (hand supine, robotic static, hand vertical, robotic centrifugation)

The measured CCA average diameter (*D*_*CCA*_) for manual ultrasound in supine position are 7.1 ± 0.2 mm, the vessel wall distension (*∆D*_*CCA*_) 565 ± 134 μm. For robotic sonography without centrifugation *D*_*CCA*_ are 6.9 ± 0.3 mm and *∆D*_*CCA*_ 493 ± 120 μm and 6.7 ± 0.6 mm and 334 ± 87 μm for robotic sonography during centrifugation (Figs. 8 and 9). Centrifugation induces no significant difference in carotid diameter for all three conditions. However, the carotid distension is significantly lower during centrifugation compared to manual, and, robotic (*p* < 0.05) sonography in supine position without centrifugation (*p* < 0.05).

**Fig. 10:**
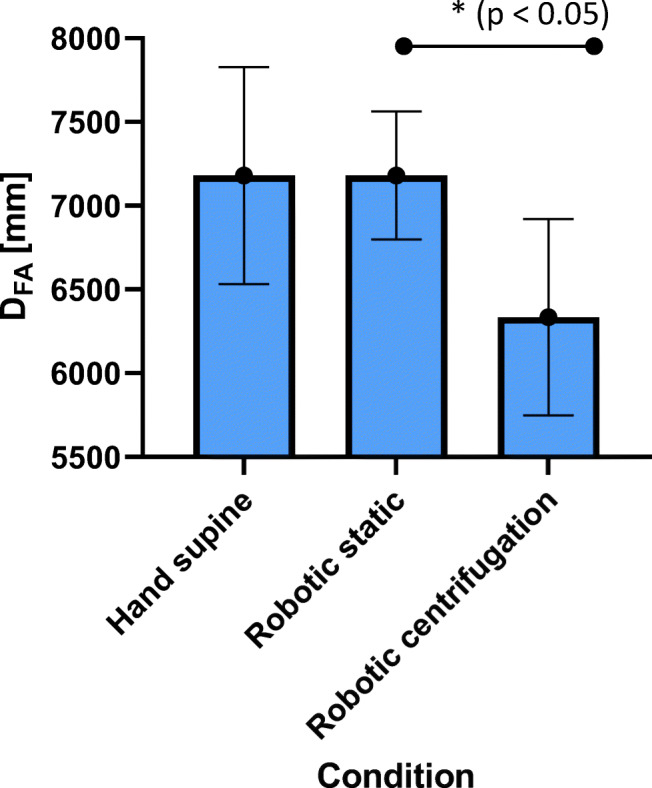
Femoral diameter (*D*_*FA*_) for all four conditions (hand supine, robotic static, hand vertical, robotic centrifugation)

**Fig. 11:**
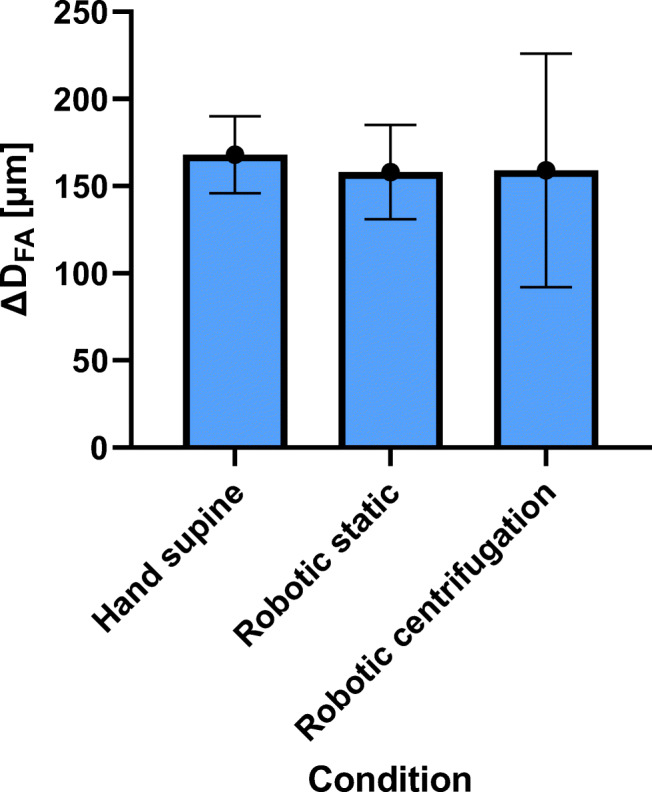
Femoral distention (*∆D*_*FA*_) for all four conditions (hand supine, robotic static, hand vertical, robotic centrifugation)

**Fig. 12:**
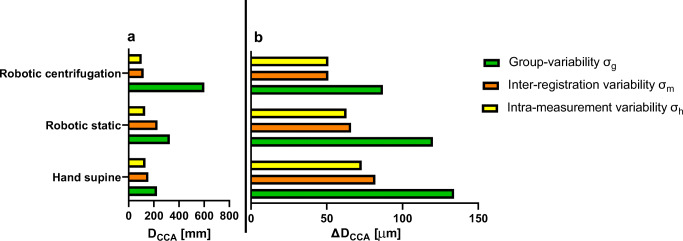
Variability of **a**: carotid diameter measurements (***D***_***CCA***_) [mm] and **b**: carotid distension (***∆D***_***CCA***_) [μm] for the three different measurement conditions

**Fig. 13:**
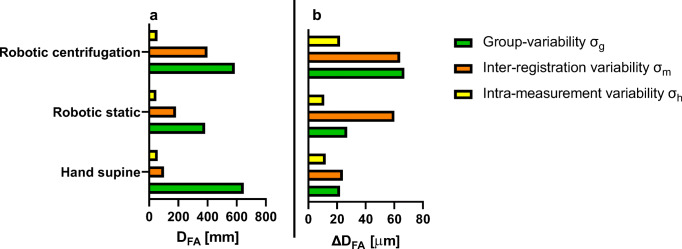
Variability of **a**: femoral diameter measurements (*D*_*FA*_) [mm] and **b**: femoral distension (*∆D*_*FA*_) [μm] for the three different measurement conditions

For femoral artery average measured diameter (*D*_*FA*_) and distension (*∆D*_*FA*_) are 7,2 ± 0.6 mm and 168 ± 22 μm for manual ultrasound in supine position, 7.2 ± 0.4 mm and 158 ± 27 μm for robotic sonography without centrifugation, and, 6.3 ± 0.6 mm and 159 ± 67 μm for robotic sonography during centrifugation (Figs. 10 and 11). The decrease in diameter during centrifugation is significant compared with robotic sonography, however it does not reach significance with manual ultrasound in supine without centrifugation (*p* = 0.1). No significant difference in distension could be measured in any condition Figs. 12 and 13.

For both the *D*_*CCA*_, and for *D*_*FA*_, the intra-registration variability, *σ*_h_, is smaller than the inter-registration variability *σ*_m_, that is in turned smaller than the inter-subject variability *σ*_g_ (*σ*_h_ < *σ*_m_ < *σ*_g_) for the 3 conditions: hand-held, remotely controlled without and with centrifugation (Tables 1 and 2). Furthermore, the results show that for both the *D*_*CCA*_ and the *D*_*FA*_ the intra-measurement variability are not increased between hand-held and remotely controlled measurements without and with centrifugation (Tables 1 and 2).

**Table 1:** Variability of carotid diameter measurements (in μm) for the three different measurement conditions

	Hand supine	Robotic static	Robotic centrifugation
Mean Diameter	7132	6876	6714
Group-variability, *σ*_*g*_	±224	±326	±601
Inter-registration variability, *σ*_*m*_	±156	±229	±118
Intra-measurement variability, *σ*_*h*_	±133	±130	±103

**Table 2:** Variability of femoral diameter measurements (in μm) for the three different measurement conditions

	Hand supine	Robotic static	Robotic centrifugation
Mean Diameter	7180	7181	6334
Group- variability, ***σ***_***g***_	±648	±383	±586
Inter-registration variability, ***σ***_***m***_	±101	±183	±399
Intra-measurement variability, ***σ***_***h***_	±58	±49	±56

For the carotid distensibility, *∆D*_*CCA*_, the intra-registration variability, *σ*_h_, is smaller than the inter-registration variability *σ*_m_, that is in turned smaller or equal to the inter-subject variability *σ*_g_ (*σ*_h_ < *σ*_m_ ≤ *σ*_g_) for the 3 conditions: hand-held, remotely controlled without and with centrifugation (Table 3). The distensibility measurement at the femoral, *∆D*_*FA*_, do not show a consistent order among the three measures of variability (Table 4).

**Table 3:** Variability of carotid distention measurements (in μm) for the three different measurement conditions

	Hand supine	Robotic static	Robotic centrifugation
Mean Distension	565	493	334
Group- variability, ***σ***_***g***_	±134	±120	±87
Inter-registration variability, ***σ***_***m***_	±82	±66	±51
Intra-measurement variability, ***σ***_***h***_	±73	±63	±51

**Table 4:** Variability of femoral distention measurements (in μm) for the three different measurement conditions

	Hand supine	Robotic static	Robotic centrifugation
Mean Distension	168	158	159
Group-variability, ***σ***_***g***_	±22	±27	±67
Inter-registration variability, ***σ***_***m***_	±24	±60	±64
Intra-measurement variability, ***σ***_***h***_	±12	±11	±22

## Discussion

In this study, a quantitative evaluation of a telerobotic system for vascular ultrasound measurement on a short arm human centrifuge was investigated. To the author’s knowledge, hemodynamic measurements using remotely controlled ultrasound functional imaging during centrifugation were never been performed on humans. Repeated assessment of the vessel means diameter and wall distension of carotid and femoral artery showed a good feasibility under static condition, as well as under, hypergravity conditions (with +2.4 Gz at feet level) on a Short Arm Human Centrifuge. No significantly differences were obtained between the reference hand-held measurements and the one obtained using the robotic-arm in static conditions. The success rate at the carotid and femoral arteries were 71% and 43% during centrifugation. Specificity of the measurements during centrifugation could be demonstrated by the intra-measurements variability that are lower than the inter-measurement variability.

The comparison of heart rate and blood pressure values during supine baseline and centrifugation showed a significant increase due to g-load. During centrifugation increased femoral diameter were expected as a result of fluid shifts toward lower extremities. Recent findings (Verma et al. 2018; Goswami et al. 2015) indicating that centrifugation with +2 Gz at feet result in analogue responses of cardiovascular system and autonomic blood pressure control to standing in Earth’s gravity. The ultrasound measurements considered in this study correspond to values find in literature. In Leguy et al. 2009 (3), an average femoral diameter (at rest in supine) on a similar subject group (6 healthy young male) of 4.1 ± 0.5 mm is reported, while in this study this parameter equals 6.6 ± 1.0 mm. The higher average diameter and group variability for this study could be explained by a slightly difference in the measurement position (probably more proximal) and by the small sample size of the groups. The ultrasound measurement reproducibility is similar with an intra-measurement’s variability of 0.2 mm and an inter-measurement variability equals 60 μm in both studies. If converted to percentage of the diameter, the distension values for the femoral are also matching with 2.3 ± 0.2% and 2.7 ± 1.4% in this study, and, in Leguy et al., respectively.

A good reproducibility and precision of the remotely controlled vascular ultrasound assessment under centrifugation could be demonstrated. No significant differences between the measurements performed by hand in supine position and the ones obtained with the ultrasound probe attached at the robotic arm in supine position without centrifugation were found. These results demonstrate that a good positioning of the probe could be reached using the robotic arm, and, that the pressure applied to the subject skin by the probe (limited to 20 Newton) did not disturb the quality of the ultrasound measurements. Considering the inter-measurement variability *σ*_h_ as a metric for the precision of the measurements, our results show that the measurements performed with the robotic arm during centrifugation are as precise as the measurements obtained under non-moving conditions. Furthermore, a good reproducibility is reflected by the low inter-measurement variability *σ*_m_ between the repeated centrifugal runs. Overall, the expected relationship between the variability parameters, *σ*_h_ < *σ*_m_ < *σ*_g_, is met except for distension at the femoral artery (for which the capacity of functional ultrasound detection is nearly reached).

The vascular parameters could be quantitatively assessed using the robotic-arm in supine position with and without centrifugation, and, demonstrate physiological behaviour. The diameter of the carotid artery was maintained during centrifugation, that can be explained by the autoregulation of blood flow to the brain and the shear-stress regulated CCA diameter regulation. As expected, the distention of the carotid artery decreased during centrifugation following the course of the decreased pulse-pressure (the carotid artery being characterised by mainly linear-elastic walls). Furthermore, the obtained decrease in the femoral diameter during centrifugation might be explained by the vasoconstriction induced by blood pooling towards the lower extremity, as during standing (19, 20). Due to the limited sample size and short exposure to g-load these findings require further evaluation in order to rich higher level of statistical significances.

From 9 subjects that we tested in this pilot trial, one subject showed pre-syncope symptoms during centrifugation. This pre-syncope may have been caused by a vasovagal reaction that could be triggered by some additional pressure applied on the baroreceptor of the carotid artery by the remotely controlled robotic system. Being aware of this potential risk, each subject had been tested for a potential drop of heart rate when pressure is applied by the ultrasound probe at the carotid artery (close to the carotid sinus) under supine position. No subjects presented with an increased heart-rate. However, these tests could not be performed during the centrifugation (for technical as well as safety reason) therefore such a risk for pre-syncope could not be avoided. Although limited in maximal applicable pressure, the visual control alone using cameras during centrifugation is not optimal. A special attention to the first minute of centrifugation while the robotic arm is used at the carotid level is being recommended.

Potential steps to optimize a robotic controlled ultrasound system are a) distance measurement between probe and participant’s skin b) a 3-dimensional visualisation of the robotic arm (e.g. using virtual reality googles) to increase accuracy of probe positioning and c) a haptic feedback system to precisely control the applied pressure of the probe. Such technical developments would allow the imaging of internal organs (e.g. heart), which requires a dynamic and precise control of probe position and pressure during centrifugation.

We conclude that with the current state-of-the-art robotic systems ultrasound measurements during centrifugation are feasible by trained examiners for peripheral arteries as the carotid artery and femoral artery. Furthermore, the successful measurements during centrifugation demonstrated reproducibility and precision that are as good as under static conditions. A better measurement success ratio during centrifugation could be achieved by better manipulator feedback.

## Data Availability

Correspondence and requests for materials should be addressed to timo.frett@dlr.de The datasets generated during and/or analysed during the current study are available from the corresponding author.

## References

[CR1] Brands PJ, Hoeks APG, Willigers J, Willekes C, Reneman RS (1999). An integrated system for the non-invasive assessment of vessel wall and hemodynamic properties of large arteries by means of ultrasound. Eur J Ultrasound.

[CR2] Clement G (2017). International roadmap for artificial gravity research. NPJ Microgravity.

[CR3] Clément G, Pavy-Le Traon A (2004). Centrifugation as a countermeasure during actual and simulated microgravity: a review. Eur. J. Appl. Physiol..

[CR4] Dogangil G, Davies BL, Rodriguez F, Baena Y (2010). A review of medical robotics for minimally invasive soft tissue surgery. Proc Inst Mech Eng H.

[CR5] Evans CR, Medina MG, Dwyer AM (2018). Telemedicine and telerobotics: from science fiction to reality. Updat. Surg..

[CR6] Frett T, Mayrhofer M, Schwandtner J, Anken R, Petrat G (2014). An innovative short arm centrifuge for future studies on the effects of artificial gravity on the human body. Microgravity Sci Technol.

[CR7] Frett, T., et al.: DLR-AG Facilities & Research Plans. (2014b)

[CR8] Frett T, Petrat G, van Loon JJWA, Hemmersbach R, Anken R (2016). Hypergravity facilities in the ESA ground-based facility program – current research activities and future tasks. Microgravity Sci Technol.

[CR9] Goswami N, Bruner M, Xu D, Bareille MP, Beck A, Hinghofer-Szalkay H, Blaber AP (2015). Short-arm human centrifugation with 0.4g at eye and 0.75g at heart level provides similar cerebrovascular and cardiovascular responses to standing. Eur. J. Appl. Physiol..

[CR10] Gunga, H.-C., et al.: The cardiovascular system in space, in Cardiovascular System, Red Blood Cells, and Oxygen Transport in Microgravity. Springer. p. 11–34 (2016)

[CR11] Iwase, S., et al.: Effect of centrifuge-induced artificial gravity and ergometric exercise on cardiovascular deconditioning, myatrophy, and bone metabolism by simulated microgravity*.* Proceedings of Annual Meeting of the Physiological Society of Japan, 2007: p. 189–189 (2007)

[CR12] Lee SMC (2015). Orthostatic intolerance after ISS and space shuttle missions. Aerosp Med Hum Perform.

[CR13] Leguy CA (2009). Model-based assessment of dynamic arterial blood volume flow from ultrasound measurements. Med. Biol. Eng. Comput..

[CR14] Linnarsson D (2015). Effects of an artificial gravity countermeasure on orthostatic tolerance, blood volumes and aerobic power after short-term bed rest (BR-AG1). J Appl Physiol (1985).

[CR15] Marescaux J, Rubino F (2003). Telesurgery, telementoring, virtual surgery, and telerobotics. Curr Urol Rep.

[CR16] Meinders JM, Hoeks AP (2004). Simultaneous assessment of diameter and pressure waveforms in the carotid artery. Ultrasound Med. Biol..

[CR17] Simón, X., Engle, J., Clark, T.K.: Artificial Gravity System Configurations Informed by Physiological Spin-Tolerance Research, In: 2018 AIAA SPACE and Astronautics Forum and Exposition (n.d.)

[CR18] Stenger MB, Evans JM, Knapp CF, Lee SMC, Phillips TR, Perez SA, Moore AD, Paloski WH, Platts SH (2012). Artificial gravity training reduces bed rest-induced cardiovascular deconditioning. Eur. J. Appl. Physiol..

[CR19] Verma AK, Xu D, Bruner M, Garg A, Goswami N, Blaber AP, Tavakolian K (2018). Comparison of autonomic control of blood pressure during standing and artificial gravity induced via short-arm human centrifuge. Front. Physiol..

